# Teaching First Aid to Prospective Teachers as a Way to Promote Child Healthcare

**DOI:** 10.3390/healthcare9040367

**Published:** 2021-03-25

**Authors:** María del Carmen Olmos-Gómez, Francisca Ruiz-Garzón, Paula Pais-Roldán, Rafael López-Cordero

**Affiliations:** 1 Department of Research Methods and Diagnosis in Education, Faculty of Education and Sport Science, University of Granada, 52071 Melilla, Spain; mcolmos@ugr.es (M.d.C.O.-G.); paula.pais.roldan@gmail.com (P.P.-R.); 2 Department of Didactics of Social Science, Faculty of Education and Sport Science, University of Granada, 52071 Melilla, Spain; raloco@ugr.es

**Keywords:** schools, health, first aid, qualitative research, social science

## Abstract

This article aimed to analyze, through a qualitative study (i.e., semi-structured interview), the opinions and knowledge of fourth-year future teachers at a Spanish public university (University of Granada) regarding training and the need for first aid (FA) at school. With a sample of 70 subjects in their last year of training, our conclusion is that although they are aware of the importance of first aid for their professional development, there is no such training in their careers, and thus they have great difficulty understanding how to react to emergency situations on the job.

## 1. Introduction

The teaching and usefulness of first aid (FA) knowledge for the general population is a matter of growing importance. As an example, in recent years, one of the main objectives of the European Resuscitation Council (ERC) [1] was to ensure that every citizen knew and properly applied basic cardiopulmonary resuscitation (CPR). In Spain, the contents on FA are part of the official curriculum of primary education students (i.e., students aged 6–12 years) [2]. Studying the presence of FA in educational legislation in Spain shows that although these contents are included in primary education and compulsory secondary education, it is taught during Physical Education class. They are also currently found in several subjects of the curriculum [3].

However, training FA to a teaching staff (early childhood education teachers, primary education teachers, or secondary education teachers) is not required at the national level [4], and not all universities offer these contents to future teachers [5]. In fact, the literature shows that less than 30% of university degrees in primary and/or early childhood education in Spanish public or subsidized universities included FA in the published syllabus of their subjects [5]. The consequences of this deficient and unequal training have been analyzed in several studies [6,7,8,9,10], all of which agreed that FA training teachers or future teachers is scarce, especially in terms of CPR maneuvers and an automated external defibrillator (AED) usage. As such, many researchers have suggested the need for teachers to receive adequate training in these areas.

In Spain [11], the repercussions of child and school accidents show, at the European level [12], that accidents or unintentional injuries in pediatric age occur mostly at home and in schools; more than 44% of unintentional injuries that occur outside of the home happen at school, the main injuries being wounds, blows, sprains, fractures, and burns. These injuries require medical assistance in 27% of cases. Fortunately, only a minority (<2%) require hospital admission [13]. Vital emergencies and cardiorespiratory arrest (CRA) can happen anywhere, with drowning being the second leading cause of unnatural childhood death worldwide [14].

Sudden infant death syndrome and respiratory diseases are the main causes of CRA in children [15]. Although the early assistance of bystanders with CRA maneuvers can increase survival, Mpotos and Iserbyt [16] found that schoolteachers felt incompetent regarding their lack of CRA training. Although many teachers mentioned prior training, only a minority of mostly young elementary school teachers felt competent about it and were willing to teach it to their students, with the propensity of individuals to act in an emergency situation being linked to FA education, which needs to be expanded to help the learner develop the ability and willingness to help in these situations [17].

Reviewing the evidence on the effects of training in FA programs, which train to overcome inhibitors of emergency helping behavior [18], could lead to a higher rate of helping. In the quest to decrease these barriers, the effect of conventional FA training as opposed to conventional training plus supplemental training was investigated, concluding that supplemental training did not alter helping behavior [19].

For all of these reasons, the ability of schoolteachers to perform good quality basic life support is a prerequisite for participation in basic life support training for schoolchildren [20]. Thus, FA education should be extended to support the student in developing both the skill and intention to help [21]. Instruction should include practice in order to enable children to perform physical tasks involved in CPR [22].

In the case of teaching FA in infancy, the knowledge acquired by very young children trained by their own teachers in nursery school was evaluated. This population were trained by emergency medical teams to perform basic FA [23]. This study demonstrated the ability of very young children to assimilate basic skills taught by their own teachers. Children as young as 4–5 years old were able to learn and apply basic FA. The teaching of FA also led to more active helping behavior and increased empathy in children [24]. Students as young as 9 years old have been found to be able to effectively learn basic life support skills, including AED deployment, correct recovery position, and emergency call skills [25]. However, not all children achieve and maintain the recommended CPR chest compression depth of 5–6 cm [26,27]. The quality of chest compressions performed by schoolchildren improves with age, but their overall performance is poor. It has been found that 13 years old is the minimum age in which one can achieve a minimum quality of CPR similar to that of an adult [27,28,29].

The aim of this article was to analyze an assessment that targeted fourth year teachers who were FA training their students at a Spanish public university (University of Granada). The qualitative study wondered whether these teachers knew what FA consisted of, what importance they felt it held in their future teaching profession, and how they wanted to receive such training. As such, the aim was to provide current and expanded information on this subject to support the need to implement FA as a compulsory training and to guide the methodology, examining the most demanded strategies for learning and thus collaborate in promoting children’s health from school.

## 2. Materials and Methods

### 2.1. Participants

The sample consisted of interviews with students in their last year (fourth year before the end of the degree) studying early childhood and primary education at the University of Granada. It was a non-probabilistic and intentional sampling, focused on the training and information needs of the sample. All participants gave their informed consent before participating in the study; confidentiality and anonymity were guaranteed based on the Declaration of Helsinki. The study was approved by the department of social responsibility code (ML_18_2-20). The size of the sample of students enrolled in the 2020 academic year is as follows: *n* = 26 students enrolled in an early childhood education degree (37.14%) and *n* = 34 students enrolled in a primary education degree (62.85%). The total sample number was 70 student–teachers (38.6% were male and 61.4% were female). The mean age of the interviewees was 21.6 years.

### 2.2. Instrument

The instrument used to collect information was a semi-structured interview (see Table 1).

For this research, an initial ad hoc interview was developed based on previous studies (Olmos, Pais, and Tierno [5]) and adapted according to the collected information. Content validity was established via expert analysis, with the participation of 9 experts whose responses were used to determine content validity. The experts determined the clarity, coherence, relevance, and appropriateness of each interview question. Only the questions in which the experts agreed on a percentage of agreement above 87% were selected for the interview, which provided a high value of content validity [30].

During the validation process of interviews, data triangulation of the information sources was performed to establish credibility. Subsequently, data saturation was performed via consensus and monitoring interviewee reports. Transferability was obtained by interviewing the total number of students enrolled in the course. Finally, the criteria of dependence and confirmability, as indicated by Olabuénaga [31], were carried out on the basis of 7 external auditors, experts in the subject, and educational research. We examined the process of data collection and interpretation to avoid errors or biases.

### 2.3. Process

#### 2.3.1. Data Collection

The interviews were conducted during in January and February of the 2020 academic year. Each interview took 25–35 min. Prior to the interview, interviewees were given indications to better understand the interview. The interviewer clarified any doubts that arose during the process. The study was conducted according to the guidelines of the Declaration of Helsinki and approved by the Ethics Committee of Cooperation and Social Responsibility Department of the University of Granada (code ML_18_2-20).

#### 2.3.2. Data Analysis

For the analysis of the data, a content analysis was carried out using a system of a posteriori categories to code data, which was done inductively based on interview transcriptions. Three categories and seven codes were extracted (see Table 1). This allowed us to understand the student–teachers’ perception regarding the concept of FA, FA as an essential part of teacher training, innovation, and the future improvement of FA training.

The interviews were transcribed in Spanish and subsequently translated into English by a native expert. Transcription and data analysis was performed with a NVivo qualitative data analysis computer (Qualitative Software Research International (QSR International), Melbourne, Australia, 2020).

Reliability was performed via expert consensus using the Delphi technique [18], with 90% agreement in the process. All codes that were unclear or did not meet sufficient agreement were modified or deleted.

## 3. Results

### 3.1. Concept of First Aid

When the informants were asked about the concept of FA, we observed that, in general, they spoke of first actions to be taken when faced with a person in need of health care. We distinguished different nuances for the informants emphasize.

#### 3.1.1. Initial Actions

When they referred to emergency actions, they focused on accidents of any kind, whether or not the injured person’s life was in danger. These are basic skills needed to help someone with health problems. These are important actions because the future health of the patient depends on what actions are taken. Some related the concept of FA to the first basic actions in an emergency in which the aim was to control and help in emergency health situations. (For example: “Basic actions to control and help in health emergency situations”; “first basic processes to help someone.”)

Second, they considered actions prior to the arrival of a medical team. These actions are not conceivable without the supervision of a team of health experts. (For example: “The FA before you call or before the health services arrive.”; “first aid is the interventions made with a person who has a clear and evident serious health problem before the specialized health services arrive.”)

There are subjects who considered it essential to have medical supervision after FA maneuvers since they were performed without any medical materials. Further, they deemed it essential to refer the injured subject to a health center or hospital where he/she can be attended with all of the necessary resources for his/her improvement.

Other characteristics concerning the concept of FA was the solidarity action one feels when helping a person who cannot fend for themselves or who requires specific attention. (For example: “They are a form of response to quickly and effectively help a person who needs it in a serious case”; “to rescue a person before qualified personnel arrive when the person is in danger.”)

A set of action guidelines, procedures, methods, or basic and essential techniques allow one to attend to someone in an accident or any medical emergency so that injuries do not worsen. (For example: “First aid maneuvers are guidelines on how to act in the event of an emergency”; “techniques or procedures can be used to assist a victim in case of an accident.”)

For the interviewees, it was important to consider that FA is an action protocol that required a minimum amount of training in a series of techniques and procedures for various emergency situations. (For example: “It is the immediate assistance and care that must be given to a person in case of emergency or accident, while the health services arrive. I think that training in first aid is a great help for everything and that it should be done in all grades and schools.”)

For other subjects, it was not so much a matter of knowing a series of techniques or protocols for action but of having the ability to react in time without losing control in a situation where help is needed. Another aspect addressed by our informants was the seriousness or lightness of the patient receiving FA. Some referred to the intervention performed to solve minor problems such as injuries, bruises, bleeding, etc., while others referred to extremely serious situations in which resuscitation could be attempted to save a life. (For example: “First aid is the ability to know how to react to a given action at a given time”; “actions or interventions to solve a problem (e.g., bleeding, injuries, bruises, contusions, and asphyxia).”)

#### 3.1.2. The Location of Learning First Aid

The four most frequently mentioned places where informants reported to have learned FA were (1) the university, (2) high school, (3) external courses offered by city programs or non-government organizations (NGOs), etc., and (4) school. The places where less references were obtained came through the Internet, television, or the family. The FA training that informants claimed to have received at university was from seminars and voluntary courses organized by faculty members. (For example: “The first talk I had about first aid was in the last year of my primary education degree.”)

In high school, some interviewees stated that they received basic notions about FA in different subjects. (For example: “In high school, in compulsory secondary education and Physical Education subjects, we were taught some basic notions about first aid”; “in high school we talked about it in some subjects.”)

As stated earlier, they also purported to have received FA training through different institutions and organizations such as NGOs, conferences, sports clubs, etc. (For example: “In external courses, such as those offered by the Red Cross”; “external courses, such as the Spanish Federation of Rescue and Lifesaving.”)

Other informants claimed to be self-taught in FA by reading and searching for information on the Internet, or even by watching television programs dealing with these subjects. Still, these interviewees recognized the potential unreliability of some of this information. (For example: “What is shown in movies and TV series cannot be considered as reliable sources of information…”)

There was another minority group that acknowledged never having received information on FA nor remembering having received it throughout their academic or professional career. (For example: “I have never attended a first aid training course before.”)

Lastly, we found those who claimed to have been trained through relatives who were medical professionals or who had personal experience with incidents that occurred in their family environment. (For example: “In my family, my mother is a nurse”; “…with our own nieces, nephews, cousins, siblings, and neighbors, sometime something happened to them either serious or minor where first aid was required.”)

### 3.2. First Aid as an Essential Part of Teacher Training

#### 3.2.1. Purpose and Importance of First Aid Training for a Teacher

Our informants considered FA training to be essential for anyone working in direct contact with other human beings, and even more so when dealing with minors who are susceptible to accidents in the educational environment. The training of a teacher in this aspect should be, according to our informants, a mandatory requirement, and some even considered it a requirement to pass competitive examinations. In a classroom or on the playground, there are a multitude of accidents every day that require knowledge of FA and a teacher must know how to act in some of these situations. Children tend to have less sense of danger and thus engage in riskier activities, especially as they develop their vulnerabilities and sensitivities. (For example: “Totally necessary. I would even highlight it as mandatory, for both teachers and students”; “FA is of vital importance because infants and children fall a lot and get hurt playing in the playground.”)

Informants also considered FA as not basic because it requires a teacher to know how to control a situation at all times and understand what kind of attention each student requires. This necessitates a teacher to master the tension of the moment and the nervousness of the entire student body.

Our informants deemed FA training important because it enables one to confidently act when needed, reduce a problem’s severity, save lives, and give an adequate response. Teachers are first responders in many situations, which is why they demand training to know how to deal with an emergency situation. (For example: “We can never know what might happen in a classroom, and if it does happen, it is necessary to be able to reassure every student while also performing the necessary maneuvers to save their lives so as not to lose control and have something more serious happen.”)

#### 3.2.2. Frequency of a Teacher’s Use of FA in His or Her Work

For most of our informants, the frequency with which a teacher used FA in his or her center was thought to be a major part of the center, i.e., in the classroom, when doing manual work, walking the stairs, pushing and shoving by classmates, on the playground, etc. They also differentiated the type of assistance provided. The most frequently attended cases are usually falls, scratches, small cuts, etc., which occur at recess and are usually not serious incidents. More serious cases such as choking or cardiac arrest are practically nonexistent. (For example: “It occurs around once a week, although it can be on a daily basis”; “every day there are falls, blows, etc.”)

Other informants considered that there was no exact frequency to determine FA activities. Accidents can happen at any time; they are unpredictable. Children are constantly moving, are interactive, and express curiosity about everything and sometimes some actions provoke accidents. (For example: “There is no exact frequency as it does not depend on a specific factor.”)

Our interviewees qualified that the frequency itself was high in most cases. In some, they specified that it was difficult to measure, yet there was no low frequency.

#### 3.2.3. Preparation/Competence to Deal with a Situation Requiring the Application of FA in Future Teaching Work and Its Justification

In relation to preparing informants, they faced situations that required them to use FA. The greatest number of references was found for those who claimed to have basic training. Their preparation allowed them to perform basic interventions, but in more complicated or serious situations they stated that they would need more training. (For example: “To perform basic first aid I feel prepared, but if I encounter a more complicated situation then I would need more training.”)

Another group of informants recognized themselves as unprepared and believed that they should learn more. (For example: “I am not trained for a serious emergency.”)

Informants who never had the opportunity to take courses or seminars on FA recognized that they did not feel prepared at all. (For example: “I do not feel at all prepared since I have never taken a first aid class or workshop.”)

### 3.3. Innovation and Future Improvement for the First Aid Training of Future Teachers

#### 3.3.1. Beliefs about the Possible Improvement of FA Training for Future Teachers

A large number of the interviewees believed that FA training for teachers should be provided through mandatory formal education. They considered that a class on the subject should be included in the teachers’ training curriculum. This would provide teachers with more skills and a better basis to intervene safely in more diverse situations. (For example: “I want more mandatory training”; “I’d like both theory and practical training and on an annual basis.”)

Other informants thought FA training should be mandatory for teachers, but that it should not be as a subject in the degree and rather as a competence that must be accredited, just as it is done with the English language. They believed that there should be mandatory courses for all those who wanted to pursue a teaching career where they must demonstrate their theoretical and practical competences in FA. (For example: “Providing a mandatory course of several months in the afternoons for those who are going to be teachers before working in a school.”)

One group of informants believed that FA training future teachers could be improved through ongoing training and a person’s educational cycles. From infant levels, content adapted to each cycle should be implemented to bring students closer to the subject of FA. (For example: “Implementing collective training from an early age, and specifically when they are going to be teachers.”)

#### 3.3.2. The Type of FA Training Most Suitable for Training Future Teachers

Most informants thought that the most appropriate way to train FA to future teachers was via compulsory subjects in education degrees.

Those who considered that teachers should be trained in FA through obligatory subjects justified it by citing the importance of the subject matter. When a subject is compulsory, it gives a greater importance to the subject matter, as students have to work harder and study the material. Informants thought that if voluntary short courses were given, not everyone would attend the course, indicating that time would be wasted in carrying it out. (For example: “If there are voluntary short courses people will not attend because it is not something mandatory and the time invested in the realization of this would be wasted.”)

On the other hand, if it was a mandatory subject, attendance would become more necessary since students would technically be paying for the course and would thus strive to pass it.

A teacher may face any type of emergency and must be able to deal with a range of possible situations. Thus, if it is a compulsory subject, it should have a broader and denser content than a seminar or a course of only a few hours. FA training is something that many people believe should not be superficially learned in 1 day. They are contents that must be practiced, corrected, and reinforced in all possible cases that a teacher may face in the school. (For example: “Because it is not something that can be learned in one day, I believe that it should be put into practice and reinforced in all possible cases that may occur in a school.”)

Informants found it important to know how to act and deal with emergency situations. They believed that the university teaches them didactics but not how to act in case of health emergencies. (For example: “At university they teach us a lot of didactics but in extraordinary situations such as health emergencies or conflict resolution it would be more useful than other subjects.”)

Some informants thought if a compulsory FA subject could not be implemented in a teacher’s curriculum, it should at least be reflected as an elective subject. They considered that the courses and seminars that are taught do not make clear the theoretical nor practical contents; a subject in a regulated training would offer this adequate training. (For example: “In this way, as it is a prolonged time, the concepts are retained and internalized better than receiving them in one-hour lectures.”)

## 4. Discussion

An increasing number of studies have shown that, from an early age, children can learn basic notions of FA and basic life support, such as the development of the will to help [32] and basic resuscitation techniques [33]. In Spain, primary school students include FA as mandatory content in their school curriculum; however, at the university, this content is not mandatory for future teachers [2,4]. According to previous studies [34], teachers should be properly trained by people who specialize in FA. Herein, we interviewed future teachers, many of whom demanded specific FA training adapted into the school context so that they could better serve and train their students.

According to our results, the definition of FA is neither clear nor unanimous among participants. Most of the interviewees agreed that recognizing FA as immediate and basic actions provided in the event of an accident or alteration in the health of a person, and that these events should be followed by specialized care. However, there are certain nuances in which they differed. Some respondents included the need to receive a minimum amount of training in order to apply FA. Others focused on the importance of the time factor and control of the situation. Knowing how to react quickly in an organized manner was considered important. There were also discrepancies in terms of how serious the injuries must be in order to warrant FA (some focus on minor injuries and others on extremely serious situations).

These differences are interesting because they provide data and perceptions of concept not valued in other recent studies.

Regarding the place where people received FA training, most confirmed received it during their time at university (through lectures or seminars) and in high school (as part of a subject’s syllabus). This shows the recent effort on the part of the University of Granada to begin training future teachers in this subject. Those who refer to having knowledge through TV (series or movies) or the Internet also questioned reliability of these sources, demonstrating their ability to identify inadequate information sources. Only a minority reported not having received any training in this regard.

Coinciding with other previous studies [6,7,8], every participant considered FA training as an essential and necessary requirement for any individual working in direct contact with other people and even more so if it involves contact with children. They justified the latter with the fact that children are more vulnerable and have a lower perception of danger than adults; as such, they can occasionally assume risky behavior. Expanding on the existing bibliography, many participants demanded extensive training (and not only basic) to help them not only learn appropriate FA measures but also to control potentially dangerous situations while other students are present. A large majority of informants added that FA training should be a prerequisite for passing competitive examinations (as is the case in France).

Almost two-thirds of responders described the frequency with which a teacher dealt with situations where FA was needed as “a lot,” though they qualified that it was difficult to give an exact frequency, as these events are “unpredictable.” Here, they emphasized the view that accidents, in general, are “not very preventable,” a view that should be worked on to reduce possible avoidable risks. In fact, in recent years, the term “accidents” has been changed to “unintentional injuries” to correct the idea that they are situations caused by chance or on which no action can be taken. Here again, it is useful to have an official national registry of school accidents to detect the prevalence and incidence of “unintentional injuries” at school and possible preventive measures.

Supporting the results of previous studies [12,13], our participants agreed that the vast majority of injuries occurring at school are not serious (e.g., falls, scratches, and blows). The cases identified as serious (e.g., choking and loss of consciousness) are almost nonexistent, although they could occur whenever.

Coinciding with previous studies, the participants reported feeling unprepared. Most of our participants thought that they had a basic level of preparation to deal with minor situations, but felt unprepared to deal with serious situations. The difference in feeling more prepared seems to lie in practical experience or practical preparation. Many attempts have been made to formulate valid, effective, and safe recommendations for determining FA training techniques and procedures [4,32]. There are educational materials for preschool and elementary school teachers on FA procedures in schools [33].

However, in studies to assess the knowledge and attitudes of elementary school teachers about FA [34], the results showed that teachers had insufficient FA knowledge.

After evaluating the FA knowledge possessed by primary and preschool teachers, we determined that these contents should be included in school curricula because they are necessary [35]; young people are aware of the need to practice, repeat, and update their FA skills [36]. Efforts should be made to avoid theoretical education and transform training into something more practice-based, especially when preparing people for emergency situations [37]. The results derived from our study showed that there has been scarce FA training of early childhood education and primary education teachers [38] despite the fact that teachers are the first and foremost responsible for the safety and health in classrooms. For special education teachers, significant differences were found in the knowledge of FA depending on interest and experience [17,39].

In short, future teachers consider theoretical training to be essential but, even more so, desire practical training and implementation (i.e., simulating real situations appropriate to their context) in order to act safely, especially in serious situations involving fatal consequences and major responsibilities.

Similar to Abelairas [35], we found that the vast majority of our participants stated that they wished FA training was mandatory while they were enrolled at university. The two ideas and proposals for integrating FA training into college curricula are interesting: a subject integrated into the official curriculum (and, therefore, qualifiable) or through compulsory courses (the result of which must be apt, but not qualifiable for the university curriculum). However, both options seek to make FA training compulsory because this subject deserves to be treated as important. Further, all students should have access to this training because the contents should be deep and time consuming, followed by practice and reinforcements of skills and concepts. If FA training was offered on a voluntary basis, attendance would be lower and people would spend less time mastering the subject. A group of participants stated that even if it was not possible to implement FA training as a compulsory subject, training should at least be an optional subject, since lectures or seminars are too short to allow students to internalize content that requires time to understand.

Our findings agree with previous studies that support the idea that FA training should be a part of the university curriculum for students studying to be teachers. In addition, our findings offer interesting nuances that could help establish the basis for implementing this training. However, the present study does have certain limitations, among which we point out the selection of participants for convenience. For future research, we propose the extension of the sample with a quantitative study, as well as the extension of the sample and quantitative study at the national and European level.

## 5. Conclusions

The health of schoolchildren is an issue of growing concern not only with regard to their care in the event of an accident at school but also for training teachers in FA. According to our review, to improve both aspects, teachers must be adequately trained in FA and basic life support because they are predisposed to help and should feel confident in doing so. This requires tailored FA training.

After analyzing the various interviews, we concluded that the concept of FA should be clarified especially as to what type of injuries and severity it involves, as well as who can apply it. According to the responses obtained, FA was considered greatly important for any profession that involves contact with other people, especially children. FA education should be a part of a teacher’s training in order to help develop their ability and willingness to help in situations requiring FA [22]. FA instruction should include practice to enable children to perform basic FA tasks [23], as FA training can be done from an early age and school is a good place for this. Most participants agreed that although most injuries treated at school are minor and frequent, their preparation is still insufficient, causing them to feel insecure. Thus, they assumed that they would not be able to adequately treat more serious situations. For this reason, they demanded mandatory FA training from the university, with a high practical content adapted to their context, thus allowing them to adequately attend to health situations and provide them with strategies and skills to control situation during these events.

## Figures and Tables

**Table 1:** Categorization system, coding, and frequency.

Categories	Coding	Frequency
First aid concept	First basic actions	64
	Place of learning about first aid (FA)	59
2. First aid as an essential part of teacher training	Purpose and importance of first aid training for a teacher	61
	Frequency of a teacher’s use of first aid at work	57
	Preparation/competence to deal with a situation requiring the application of first aid in future teaching work and its justification	62
3. Innovation and future enhancement of first aid training for future teachers	Beliefs about the possible improvement of first aid training for future teachers	65
	Type of training most suitable for training future teachers in first aid	52

## Data Availability

The data presented in this study are available on request from the corresponding author.
